# Optimal Control and Optimization of Grid-Connected PV and Wind Turbine Hybrid Systems Using Electric Eel Foraging Optimization Algorithms

**DOI:** 10.3390/s24072354

**Published:** 2024-04-07

**Authors:** Saad A. Mohamed Abdelwahab, Ali M. El-Rifaie, Hossam Youssef Hegazy, Mohamed A. Tolba, Wael I. Mohamed, Moayed Mohamed

**Affiliations:** 1 Electrical Department, Faculty of Technology and Education, Suez University, Suez 43221, Egypt; 2 College of Engineering and Technology, American University of the Middle East, Egaila 54200, Kuwait; 3 Electrical Department, Faculty of Technology and Education, Helwan University, Helwan 11795, Egypt; hossamyh@hotmail.com (H.Y.H.); waelibrahim785@gmail.com (W.I.M.); 4 Reactors Department, Nuclear Research Center, Egyptian Atomic Energy Authority, Cairo 11787, Egypt; matolba@ieee.org; 5 Electrical Department, Faculty of Technology and Education, Sohag University, Sohag 82524, Egypt; moayed1190@techedu.sohag.edu.eg

**Keywords:** Particle Swarm Optimization, Electric Eel Foraging Optimization, hybrid PV and wind turbine

## Abstract

This paper presents a comprehensive exploration of a hybrid energy system that integrates wind turbines with photovoltaics (PVs) to address the intermittent nature of electricity production from these sources. The necessity for such technology arises from the sporadic nature of electricity generated by PV cells and wind turbines. The envisioned outcome is an emissions-free, more efficient alternative to traditional energy sources. A variety of optimization techniques are utilized, specifically the Particle Swarm Optimization (PSO) algorithm and Electric Eel Foraging Optimization (EEFO), to achieve optimal power regulation and seamless integration with the public grid, as well as to mitigate anticipated loading issues. The employed mathematical modeling and simulation techniques are used to assess the effectiveness of EEFO in optimizing the operation of grid-connected PV and wind turbine hybrid systems. In this paper, the optimization methods applied to the system’s architecture are described in detail, providing a clear understanding of the intricate nature of the approach. The efficacy of these optimization strategies is rigorously evaluated through simulations of diverse operating scenarios using MATLAB/SIMULINK. The results demonstrate that the proposed optimization strategies are not only capable of precisely and swiftly compensating for linked loads, but also effectively controlling the energy supply to maintain the load’s power at the desired level. The findings underscore the potential of this hybrid energy system to offer a sustainable and reliable solution for meeting power demands, contributing to the advancement of clean and efficient energy technologies. The results demonstrate the capability of the proposed approach to improve system performance, maximize energy yield, and enhance grid integration, thereby contributing to the advancement of renewable energy technologies and sustainable energy systems.

## 1. Introduction

New and renewable energy (NRE) represents a category of energy sources that have garnered increasing attention in recent times owing to their sustainability, environmental advantages, and potential to diminish reliance on fossil fuels. These energy sources are termed “new” as they have emerged as viable alternatives to traditional fossil fuels, while being “renewable” since they are naturally replenished and theoretically can be harnessed indefinitely without depleting finite resources. However, the suitability of an NRE source depends mainly on its location, and not all regions possess equal access to these resources. The transition towards NRE necessitates substantial investment in infrastructure, including the establishment of wind farms, solar arrays, and upgrades to the grid system [1].

Renewable energy sources are pivotal to tackling climate change, reducing pollution, and ensuring a sustainable energy future. Governments, businesses, and individuals are increasingly adopting these technologies to create a cleaner and more secure energy landscape for generations to come. The future of renewable energy research holds the promise of breakthroughs in next-generation technologies, improved energy storage solutions, and innovative policies that accelerate the transition to a sustainable energy future. Through collaborative efforts across borders and disciplines, researchers strive to unlock the full potential of renewable energy sources to power a cleaner, more sustainable world [2]. 

The effectiveness of a grid-connected hybrid system depends heavily on factors such as cost, reliability, and greenhouse gas reduction. To meet the energy needs of rural areas, grid-connected hybrid PV and wind turbine systems undergo multi-objective optimization. This optimization aims to maximize the renewable energy fraction as a primary objective, while simultaneously minimizing the loss of power supply probability and the cost of energy under diverse weather conditions [3]. 

PV/wind systems rely only on renewable energy sources, and the utility grid is the most dependable system available. On the other hand, surplus electricity generated by solar PVs and wind power is sold to the grid at a specified rate if it surpasses the load requirements. Numerous studies have illustrated that when extra energy is used in this manner, the LCOE is decreased [4]. 

The originality and main contribution of this study lies in its innovative approach to enhancing the performance of grid-connected hybrid PV and wind turbine systems. This paper introduces a novel perspective by analyzing the PI controller and employs two distinct optimization algorithms, namely PSO and EEFO. 

The key aspects of originality and contribution can be summarized as follows:

- The use of two optimization algorithms, PSO and EEFO, contributes to the originality of the study. Comparing and contrasting the performance of these algorithms in the context of hybrid PV–wind turbine systems provides valuable insights into their respective strengths and weaknesses.
- The unique application of EEFO specifically for optimizing hybrid systems marks a distinctive aspect of this study. EEFO draws inspiration from the foraging behavior of electric eels, introducing a novel perspective to system optimization. This approach potentially offers advantages in certain aspects of control that may not be addressed by traditional optimization methods.
- This paper provides an explanation for the optimization performance of EEFO within the context of hybrid PV–wind turbine systems. This explanation likely delves into how EEFO’s foraging-inspired mechanisms contribute to more effective and adaptive solutions in the optimization process.

This article is divided into six main sections. The introductory section outlines the problem and study contributions, and the second part highlights the recent related research work. The third section introduces a comprehensive depiction of the proposed system, elucidating its intricacies and components. Subsequently, the fourth section delineates the methodologies employed for the proposed algorithms. Section 5 is dedicated to presenting and scrutinizing the simulation results of the implemented system. Finally, the sixth section concludes the study with a conclusion and potential implications. 

## 2. Review of Related Work

In ref. [5], the focus is on the development, control, and assessment of a hybrid photovoltaic (PV) and wind power generation system connected to the grid. This study specifically examines the case of the Gabel El-Zeit region in Egypt. This indicates that the generated power is intended to be integrated into the existing electrical grid, highlighting the potential for contributing to the overall energy supply. 

In ref. [6], the authors focus on the design and control challenges associated with microgrids that are primarily supplied by renewable energy sources. The proposed paper contributes to the field of microgrid technology by addressing the challenges related to the design and control of microgrids powered by renewable energy sources. It offers insights that can be valuable for researchers, engineers, and practitioners working on sustainable and resilient energy systems. 

Article [7] investigates various energy storage technologies and their appropriateness for bolstering the integration of renewable energy sources like solar and wind power into established power grids. The goal is to provide valuable perspectives on how energy storage can augment the dependability and effectiveness of renewable energy systems. This paper presents a comprehensive examination of the present status and future potential of energy storage technologies within the realm of integrating renewable energy into power grids. 

There are a lot of challenges related to optimization in our daily lives. The search for efficient solutions for optimization issues is progressively becoming a major field of study. Finding the best answer, or a good approximation among several options under specific constraints, to a given problem is known as optimization. Many optimization problems are becoming more common and complex in a wide range of engineering domains, from image processing to artificial intelligence, because of the quick development of new technology [8]. 

The PSO algorithm draws inspiration from the collective behavior of bird flocks and fish schools, aiming to replicate the observed collective intelligence and cooperation in these natural systems. Its operational mechanism involves a population of particles navigating the search space to discover the optimal solution. Each particle adapts its position by considering its individual experience and the best-known position within the entire swarm. This movement is guided by both personal best and global best information, fostering a balance between exploration and exploitation [9]. 

The process of applying the PSO algorithm is as follows: Initialize a set of PSO algorithms with randomly assigned positions and evaluate the fitness of each particle. Adjust the velocity and position of each particle by considering both its individual best-known position and the collective most-appropriate positions of the swarm. Finally, iterate through these steps until convergence or the fulfillment of a specified stopping criterion. PSO has demonstrated its effectiveness in addressing a variety of optimization challenges, including function optimization, neural network training, and real-world engineering problems [10].

In a separate study [11], the focus is on improving the seamless integration of RES into hybrid microgrids. This is achieved by employing an optimal PID controller to determine the PID gains. This paper introduces a time-domain objective function based on voltage and current errors. A comparative analysis is carried out on the results obtained from the implementation of the LAPO and PSO algorithms. The findings indicate that the devised LAPO algorithms exhibit superior performance compared to traditional PSO, particularly in terms of input and output current, voltage, and power.

The system examined in [12] integrates both PV and wind energy sources alongside battery storage. The primary goal of this study is to pinpoint the most advantageous configurations and parameters for the hybrid system, taking into account both economic and environmental considerations. According to the Utopia Point Solution statistics, it is noteworthy that battery storage (constituting approximately 39%) and solar energy (making up around 46%) emerge as the predominant renewable energy sources, significantly contributing to the overall energy mix. 

Article [13] delves into optimizing the design of off-grid multi-carrier microgrid, addressing challenges and considerations inherent in the design of off-grid microgrids. It explores optimization techniques tailored to the unique characteristics of the Rakiura–Stewart Island microgrid. Through numerical simulations, this study reveals that the optimal strategy not only represents a low-risk investment but also yields high returns. The proposed approach stands to provide significant cost savings for the community relying on diesel for their energy needs, amounting to a substantial 54%, inclusive of electrified space heating expenditures. 

In another study [14], the improvements to a microgrid incorporating various renewable energy sources are realized by implementing a recommended multi-case power management technique alongside multi-objective PSO. The research operates under multiple constraints, concurrently minimizing three competing objective functions: greenhouse gas emissions, levelized cost of energy, and loss of power supply probability. Through the optimization process, 4 out of the 200 non-dominated or Pareto optimum alternative solutions are selected as solutions of interest. The proposed design of the Hybrid Renewable Energy Microgrid is also evaluated for effectiveness, providing valuable insights that could significantly contribute to the electrification of the targeted rural agricultural area. The findings of this research provide a wealth of information that may guide sustainable energy planning and development in the region [14].

In ref. [15], the Adaptive Sine-Cosine Algorithm (ASCA) is introduced to enhance the search capabilities of the conventional Sine Cosine Optimization (SCA) and alleviate its tendency to stagnate at local optima. ASCA achieves this improvement by incorporating modifications to the traditional SCA through the application of Levy flight distribution and adaptive operators. The Hybrid Renewable Energy System (HRES) comprises three sources: a PV source, a wind turbine, and battery storage. This study’s results confirm a substantial improvement in the performance of the HRES through the optimization of its controllers using ASCA. This enhancement has been observed under various operating conditions, including solar irradiation, temperature variations, and wind speeds. The utilization of ASCA in optimizing HRES parameters is examined in ref. [15]. 

The primary focus of [16] revolves around harnessing the synergy of solar and wind energy sources to optimize overall system performance. The aim is to provide insights into current advancements in this field, fostering the development and adoption of efficient and sustainable renewable energy solutions. The research involves evaluating various hybrid models that integrate PV and wind systems, delving into the modeling aspects and proposing enhancements for the integrated system. Furthermore, this study comprehensively discusses massive management strategies for both grid-tied and standalone systems. 

In ref. [17], the authors focus on performing a sensitivity analysis of renewable energy sources, with a specific focus on wind and solar power. The goal is to determine their impact on the costs associated with hybrid power plants in the Kermanshah region. The research reveals that variations in solar radiation intensity significantly affect the return on capital, resulting in a reduction to 9.22 years at a specific radiation intensity. Moreover, this study performs a sensitivity analysis of wind intensity, identifying an optimal turbine speed of 4.99 m/s, corresponding to a cost of electricity of USD 0.93/kWh.

Through comprehensive analysis in [18], the proposed shunt-resonance fault current limiter (SRFCL) displays remarkable improvements in the dynamic behavior and transient stability of the hybrid power system. These enhancements lead to improved active power injection and superior grid voltage profiles during grid disturbances. Comparative analysis confirms the superiority of the SRFCL over both the BFCL topology and fault ride-through control schemes across various aspects. This research provides valuable insights into optimizing fault current limiters for grid-connected hybrid renewable energy systems, emphasizing their crucial role in ensuring reliable and stable operation during grid disturbances.

In ref. [19], the versatility of EEFO is extensively demonstrated through the solution of ten engineering problems and the control of a hydropower station sluice opening under accident-tripping conditions. This study highlights the algorithm’s exceptional competitiveness, promising potential, and superior performance when tackling various complex real-world issues. EEFO shows its proficiency in both exploitation and exploration, showcasing its ability to strike a balance between the two and effectively avoid local optima [19].

In ref. [20], the authors investigate the use of a hybrid wind–PV farm as a static synchronous compensator (STATCOM) to address chaotic oscillations in a two-area power system. It evaluates the system’s performance across different scenarios, including instances of a three-phase fault and variations in electric torque and reference voltage by 20%. These scenarios aim to gauge the hybrid wind–PV farm’s ability to manage chaotic oscillations under challenging conditions.

In ref. [21], the authors introduce a mixed-integer linear programming model applied using the A Mathematical Programming Language (AMPL) alongside the CPLEX linear solver. The model integrates biomass, solar, and wind power plants, incorporating biogas units fueled by cow manure, 1 kW PV modules, and 5.1 kW wind turbines. Through result analysis, this study highlights the superiority of a hybrid approach utilizing wind, PV, and biomass energy resources for electricity generation in rural areas over singular wind turbine operations or alternative combinations of renewable energy sources.

Table 1 illustrates the key features of the present study in comparison to select previous research efforts. Upon reviewing the methods outlined in Table 1, it becomes evident that this study is the first to employ EEFO technology within grid-connected PV and wind turbine hybrid systems. 

## 3. System Description

**Figure 1:**
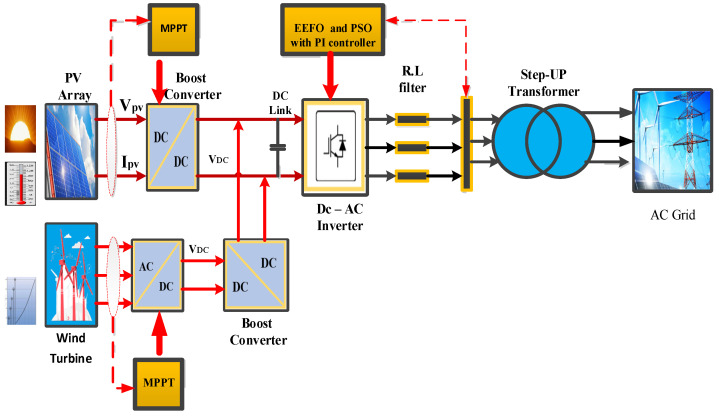
Hybrid PV/wind renewable energy generation system.

A hybrid PV/wind system is presented in Figure 1, consisting of a wind energy system, a PV energy system, a DC–DC converter, a DC link, a three-phase inverter, a step-up three-phase transformer, and the grid. This paper suggests using PSO and EEFO methods for controlling the maximum power point (MPP) and inverter PI control, respectively. The wind generator incorporates a permanent magnet synchronous generator (PMSG) and a diode rectifier for AC–DC conversion. The three-phase voltage source inverter (VSI) is linked through an RL filter to inject high-quality current into the utility grid, concurrently mitigating current harmonics at the inverter output. The outcomes of the algorithms yield values for current, voltage, and power [22].

### 3.1. Modeling of Photovoltaic Cells Mathematically

Solar radiation, ambient temperature, and PV manufacturing data serve as inputs for modeling to estimate the hourly energy output from PV panels. The influence of solar radiation and PV cell temperature on photovoltaic energy is well established [23].

**Figure 2:**
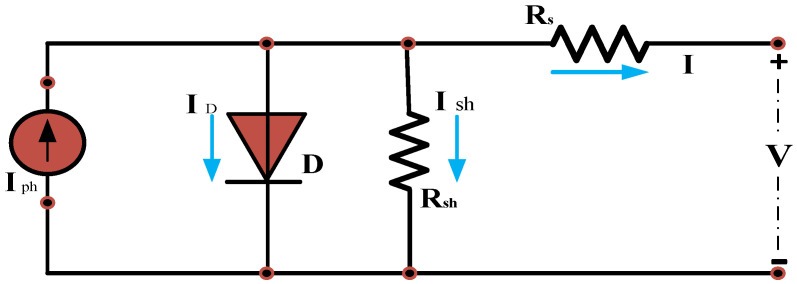
PV cell equivalent circuit.

Figure 2 depicts the critical step of mathematical modeling for PV cells, a fundamental aspect of the analysis and design of PV control systems. Equations (1)–(4) presented below define the mathematical model for PV, outlining the correlation between the voltage and current of the PV cell [24].

(1) $$I_{c}=I_{ph}-I_{o}\left\{\left.e^{\left[\frac{q}{AkT}(V_{c}+I_{c}R_{s})\right]}-1\right\}\right.$$

(2) $$V_{c}=\frac{AKT}{q}ln\left(\frac{I_{ph}+I_{o}-I_{c}}{I_{o}}\right)-I_{c}R_{s}$$

(3) $$I\;=\;I_{ph}-I_{o}\left\{\left.e^{\left[\frac{q}{n_{s}AkT}(V+n_{s}IR_{s})\right]}-1\right\}\right.$$

(4) $$V=\frac{n_{s}AKT}{q}ln\left(\frac{I_{ph}+I_{o}-I}{I_{o}}\right)-n_{s}{IR}_{s}$$

In these equations, *I* represents the current generated by the PV module, *I_c_* represents the PV cell output current, *V_c_* represents the PV cell output voltage, *I*_0_ is the diode’s saturation current, *I_Ph_* is the current generated from photons, *R_s_* is the series resistance in ohms, *R_Sh_* is the parallel resistance in ohms, *A* is the ideality factor, *K* is the Boltzmann constant, q is the charge of the electron, and *T* is the temperature in Kelvin.

### 3.2. Boost Converters with Mppt

**Figure 3:**
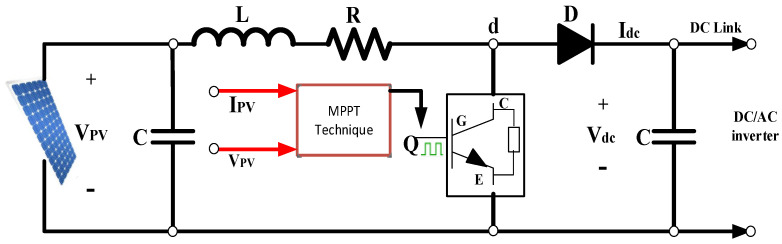
Boost converter circuit diagram.

The electrical circuit of the boost converter is illustrated in Figure 3. It is called a boost converter due to its output voltage exceeding the input. It comprises essential components including an inductor (L) with internal resistance (R), a diode (D), a DC-link capacitor (C), and an insulated gate bipolar transistor (IGBT) switch. The boost DC–DC converter aims to elevate the PV output voltage, coordinating its voltage level with that of the electrical grid via a DC/AC inverter.

In the detailed model, the boost converter amplifies the voltage from 280 V to 500 V. Employing MPPT technology in this model allows for the optimization of energy production from the PV array. This technology automatically adjusts the duty cycle to achieve the desired voltage, thereby ensuring maximum energy harvest.

In addressing certain drawbacks of the perturb and observe (P&O) method, iterative control (IC) has been proposed as a potential remedy. This includes mitigating issues such as steady-state inaccuracy and convergence speed. Leveraging the principles of disruption and observation, IC is frequently regarded as a superior technique, attributed to its evident advantages, such as a rapid and straightforward response to dynamically changing solar irradiances [25].

### 3.3. Wind Turbine

Predominantly employed in small-scale generating systems, particularly within wind power setups, the PMSG incorporates magnets on its rotor and a stator core wound in three phases. A distinctive feature of the PMSG is its ability to avoid the mechanical generation of a magnetic field, rendering it an attractive choice for renewable energy applications. Renowned for its reliability, simplicity, and robust construction, the PMSG operates efficiently without the need for a separate DC power source in its excitation circuit [26]. 

Compared to alternative generator types, the PMSG produces less mechanical friction as it lacks brushes, eliminating the requirement for a gearbox. Despite its lightweight nature, this generator exhibit an impressive power-to-weight ratio. Noteworthy is the absence of a need for a reactive magnetizing current for excitation, setting it apart from induction generators. However, it is essential to recognize that the cost of the permanent magnets essential for the PMSG can pose a constraint. Furthermore, the magnetic strength of these magnets diminishes at elevated temperatures [27].

(5) $$p_{Tu}=\frac{1}{2}\pi r^{2}\rho C_{p}\left(\beta *\lambda \right)V^{3}$$

(6) $$\lambda =\frac{{\omega m}^{r}}{V}$$

(7) $$T_{m}=\frac{1}{2}\rho AC_{p}\left(\beta *\lambda \right)v^{3}$$

where $p_{Tu}$ represents the power generated by the wind turbine, ρ is the air density in kilograms per cubic meter, *R* signifies the blade radius in meters, *C_p_* denotes the performance coefficient of the wind turbine (*C_p_*) based on the rotor pitch angle *β* (in degrees), *V* represents the wind speed in meters per second, λ indicates the tip-speed ratio, ωm represents the rotor speed in radians per second, r is the length of the blade in meters, and T_m_ corresponds to the output torque.

### 3.4. Proposed Controllers on the Three-Phase Inverter

The comprehensive control system described involves four simultaneous steps to regulate the operation of the power system. Firstly, the controller utilizes Park’s transformation, which is a mathematical technique, to convert stationary reference frame voltages and currents into corresponding rotating reference frame values. This transformation allows for more efficient control in systems with rotating electrical machines, such as synchronous machines and permanent magnet motors. Once the transformation is applied, the resulting currents (*I_q_* and *I_d_*) are compared with the commanded references. This step ensures that the actual currents align with the desired values set by the controller. In the second control loop, the system regulates the AC voltage, determining the size of the AC voltage from the voltage source inverter (VSI). Here, the reference current (*I_qref_*) is set to zero, optimizing the power supplied to the grid and ensuring that the VSI operates at unity power factor. Operating at a unity power factor means that the VSI delivers real power to the grid without causing any reactive power flow. This is crucial for the efficient and stable operation of the power system [28].

**Figure 4:**
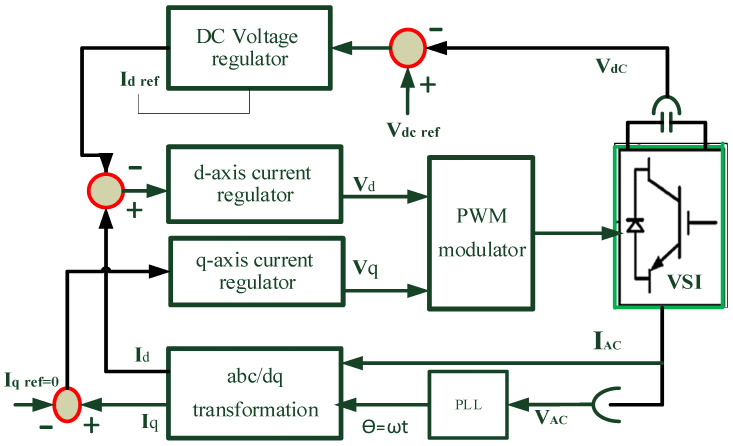
Controller block diagram for the inverter of a system.

Figure 4 illustrates the concept of operating the VSI at unity power factor. By maintaining the reference current at zero and optimizing the power supply to the grid, the control system aims to maximize the efficiency and stability of the overall power system operation. An additional crucial aspect pertains to the AC/DC VSI used for integrating the HRES. In this context, a phase-locked loop (PLL) is employed for frequency sensing, generating an angle (*θ*) that ensures synchronization among regulators and electronic switches. This synchronization facilitates the supply of power to the main grid. The grid voltage is represented by stationary frame components (*α* and *β*), and the transformed *dq* terms are provided in Equations (8) and (9), with *θ* representing the phase angle of the grid voltage.

(8) $$V_{d}=V_{\alpha }Cos\theta +V_{\beta }sin\theta$$

(9) $$V_{q}=-V_{\alpha }sin\theta +V_{\beta }cos\theta$$

The power supplied to the grid can be determined by:

(10) $$P_{g}=\frac{V_{g}\;I_{g}}{2}(1-cos\;2\;\omega t)$$

The active and reactive power output of the inverter can be determined using Equations (11) and (12).

(11) $$P=\frac{1}{2}(V_{d}i_{d}+V_{q}i_{q})$$

(12) $$Q=\frac{1}{2}(-V_{d}i_{d}+V_{q}i_{q})$$

In this transformation, achieving control solutions is complex as it requires the generation of orthogonal signals to produce real and imaginary grid current components [11,28].

## 4. Proposed Optimization Techniques

### 4.1. Particle Swarm Optimization (PSO)

The PSO algorithm operates in two main variants: the global version and the local version. In the global variant, each particle within the swarm simultaneously tracks two positions: its own optimal position (*p_best_*) and the optimal position of the entire swarm (*g_best_*). This means that each particle maintains a dual focus, aiming to find the best solution for itself while also considering the best solution achieved by the entire swarm. By doing so, the global variant encourages exploration of the search space while also exploiting the collective knowledge of the entire swarm. Conversely, the local variant of the PSO algorithm has each particle tracking its own optimal position (*p_best_*), as well as the optimal position (*n_best_*) of all other particles within its specified topological neighborhood. In this approach, particles are grouped into neighborhoods, and each particle focuses on the best solution found within its neighborhood. Unlike the global variant, which emphasizes the overall optimal position of the entire swarm, the local variant encourages collaboration and information sharing among neighboring particles. This localized approach can sometimes lead to faster convergence and better exploitation of promising regions of the search space. Equation (13), representing the velocity update, was adjusted for the local version to take the following form:

(13) $$V_{i,t+1}^{d}=\chi (V_{i,t}^{d}+{Ø}_{1}*rand\left(P_{i,t}^{d}-X_{i,t}^{d}\right)+{Ø}_{2}*rand*(P_{g,t}^{d}-X_{i,t}^{d})$$

(14) $$V_{i,t+1}^{d}=\omega *V_{i,t}^{d}+c_{1}*rand\left(P_{i,t}^{d}-X_{i,t}^{d}\right)+c_{2}*rand*(P_{l,t}^{d}-X_{i,t}^{d})$$

where $p_{l}$ is the ideal location inside the community, ${\;V}_{i}$ is the velocity vector, $P_{i}$ is the individual’s optimal position (i.e., the optimal position that the particle has experienced), and $P_{g}$ is the swarm’s optimal position (i.e., the optimal position that any individual in this swarm has experienced).

**Figure 5:**
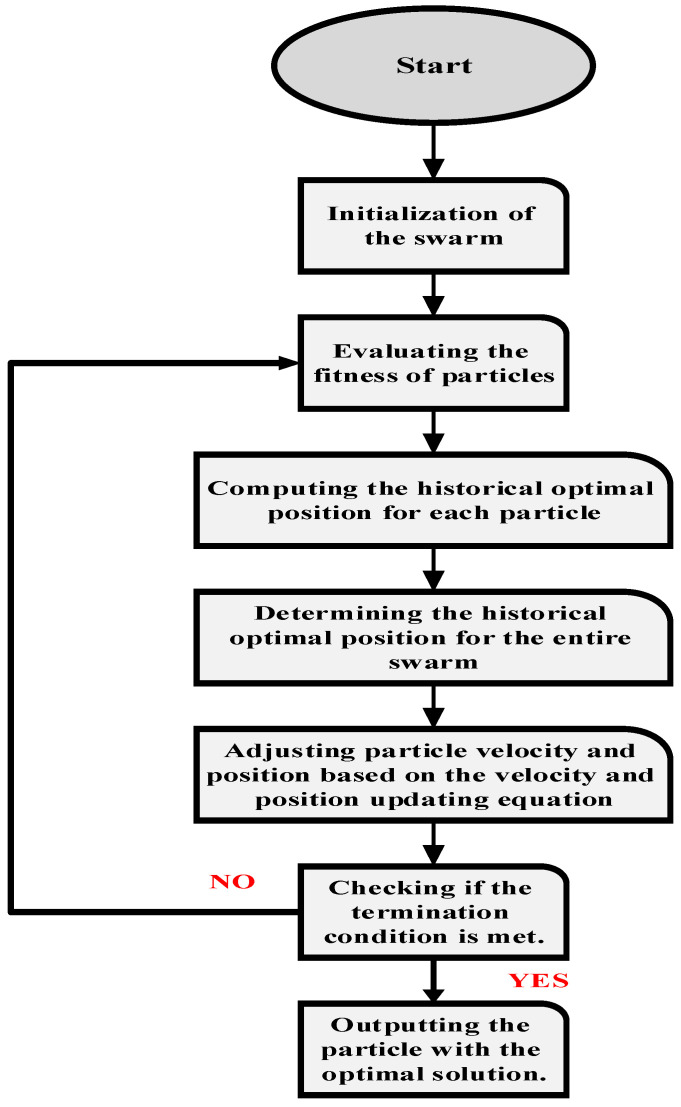
The flowchart of PSO algorithm.

The goal functions establish a distinct relationship among different aspects within the problem space. Empirical evidence underscores the effectiveness of this method as an optimization tool. The PSO algorithm flowchart is depicted in Figure 5 [10,29]. 

**Figure 6:**
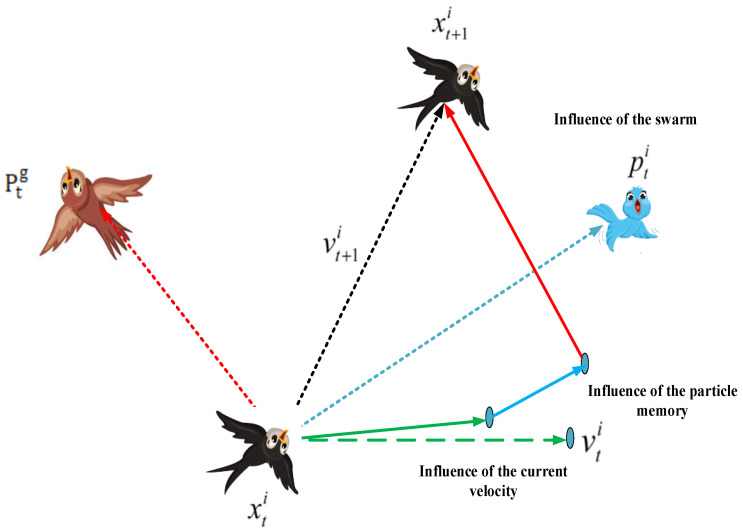
Iteration scheme of the particles.

As illustrated in Figure 6, a sociological perspective on the velocity update formula reveals that the particle’s prior velocity significantly influences the initial part of the calculation. This suggests that the particle moves inertially based on its current level of confidence.

Contemporary research on the PSO algorithm theory primarily centers around understanding its fundamental principles, including the dynamics of particle interactions and the factors contributing to its effectiveness in certain optimization problems. Investigations in this domain can be categorized into three main areas:

The first category involves examining the trajectory of an individual particle in motion.

The second category focuses on understanding the convergence behavior of the algorithm.

The third category delves into the distribution and evolution of the entire particle system over time [29].

### 4.2. Electric Eel Foraging Optimization (EEFO)

**Figure 7:**
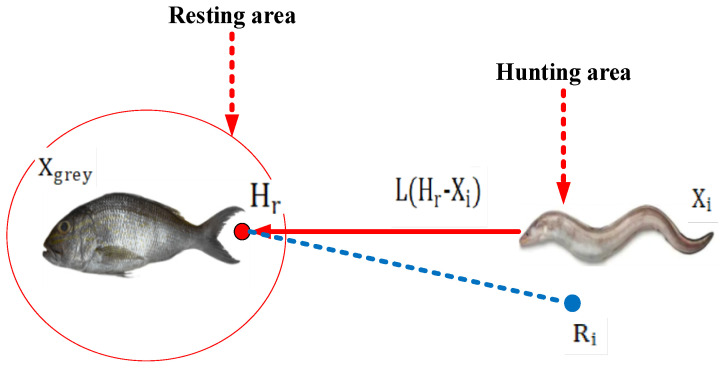
An illustration of the migration pattern.

**Figure 8:**
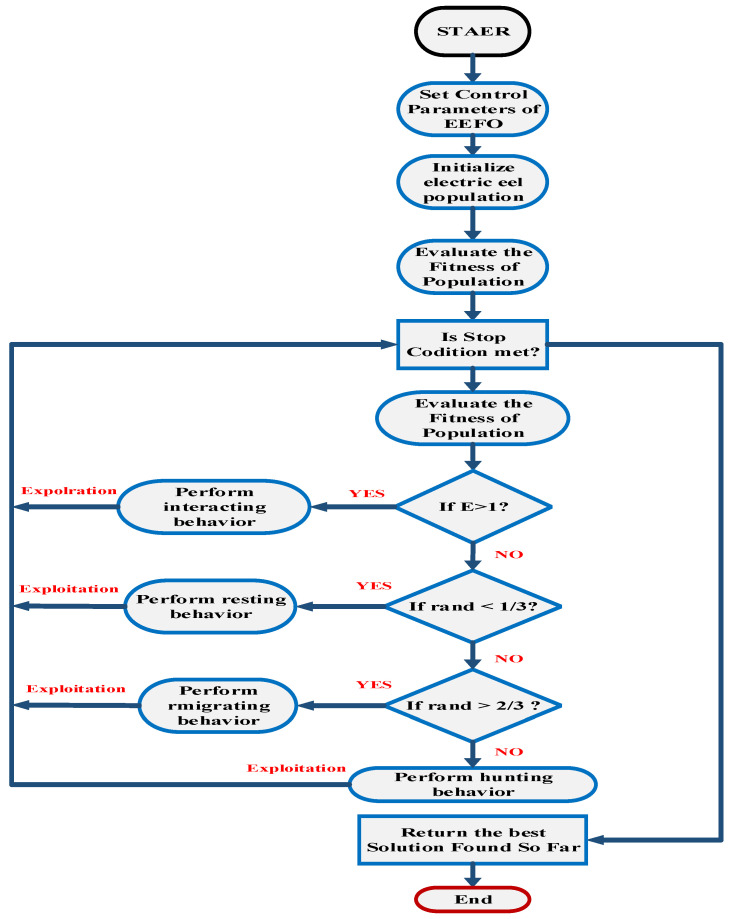
Displays the flowchart of EEFO.

EEFO begins by initializing control parameters such as the maximum number of iterations and the size of the electric eel population. A set of eels is generated randomly, and each eel employs interactive behavior for exploration when the energy factor (E) is greater than 1. During resting, migrating, or hunting, each eel engages in exploitation with the same probability when the energy factor (E) is less than or equal to 1. Candidate solutions are generated and compared with existing ones, with the best solution being updated iteratively. As the iteration progresses, the energy factor (E) decreases, prompting a transition from exploration to exploitation. The interactive process continues until a specified stopping condition is met, and the best solution is saved at that point. The flowchart and pseudo-code of EEFO are presented in Figure 7 and Figure 8, respectively [19].

Through rigorous testing, EEFO has demonstrated superior performance compared to other optimization algorithms in terms of exploration, avoidance of local optima, and exploitation. Its versatility extends to various optimization problems, including those with numerous restrictions and variables. Notably, EEFO considers immoral aspects of problem-solving. The migrating behavior of an eel is illustrated in Figure 7. Eels, equipped with the ability to perceive prey positions through low electric discharge, dynamically adjust their positions. In the foraging process, if an eel senses the approach of prey, it moves to a candidate position; otherwise, it remains in its current position [19,30].

(15) $$X_{i}(t+1)=\left\{\begin{array}{c} X_{i}(t)\;\;\;fit(X_{i}(t))\leq fit(v_{i}(t+1))\\ v_{i}(t+1)\;\;\;fit(X_{i}(t))>fit(v_{i}(t+1)) \end{array}\right.$$

where $fit(X_{i}(t))$ is the fitness of the candidate position of the ith electric eel, $X_{i}$ is the position of an eel chosen randomly from the current population, and $v_{i}$ is the position of a randomly selected food.

EEFO initialization involves specifying control parameters such as the maximum number of iterations and the size of the electric eel population. A randomly distributed set of eels is then generated. During each iteration, when the energy factor E is greater than 1, each eel engages in exploration using its interactive behavior. Conversely, when the energy factor E is less than or equal to 1, each eel has an equal probability of engaging in exploitation, whether it is resting, migrating, or hunting. To generate new candidate solutions, each case is applied to every eel, and these solutions are compared with existing ones. The current best solution is continuously updated throughout the iteration process. As the iteration progresses, the energy factor E diminishes, prompting each eel to transition from exploration to exploitation. This interactive process continues until the specified stop condition is met. The best solution achieved up to that point is then saved. The EEFO flowchart and pseudo-code are presented in the algorithm and Figure 8, respectively [19].

## 5. Simulation and Discussion of Results

### 5.1. Weather Condition of Solar and Wind Energy

**Figure 9:**
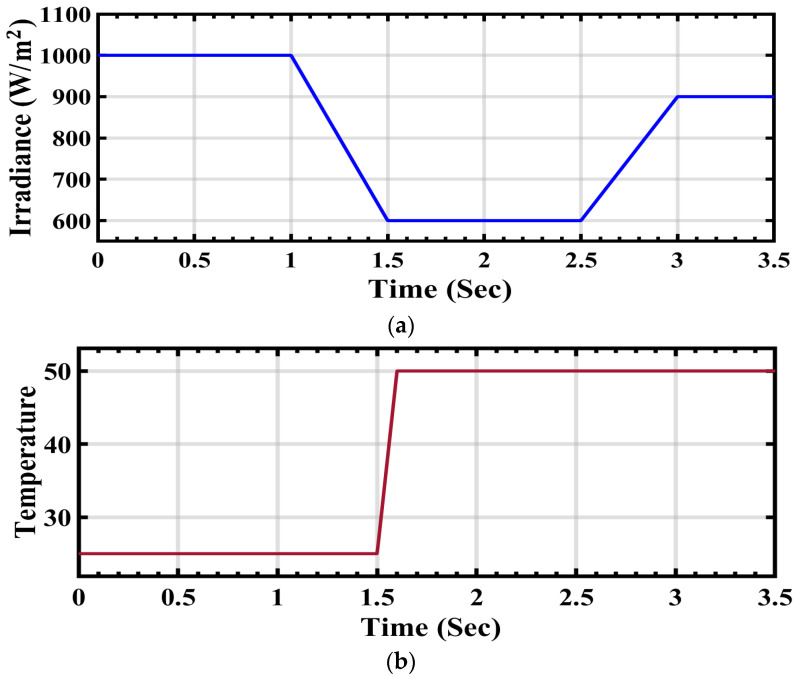
(**a**) Alterations in PV irradiation. (**b**) Temperature fluctuations for the PV array.

The system specifics are detailed in Table 2. Figure 9a depicts the step-changing solar radiation profile, which initiates at 1000 W/m^2^ and stabilizes at this value from 0 to 1 s. It then decreases to 600 W/m^2^ from 1 to 1.5 s and maintains this level until 2.5 s. At 3 s, the radiation undergoes an increase to 900 W/m^2^ until the end of the simulation. These fluctuations in radiation significantly impact the current, voltage, and energy output from the solar power station to the grid, as illustrated in the subsequent figures.

Similarly, Figure 9b illustrates the temporal variability of temperature. At the initial time of 0 t/s, the temperature is 25 °C and remains constant until 1.5 t/s. Subsequently, there is a sudden increase in temperature to 50 °C, maintaining this level until the end of the simulation at 3.5 t/s.

**Figure 10:**
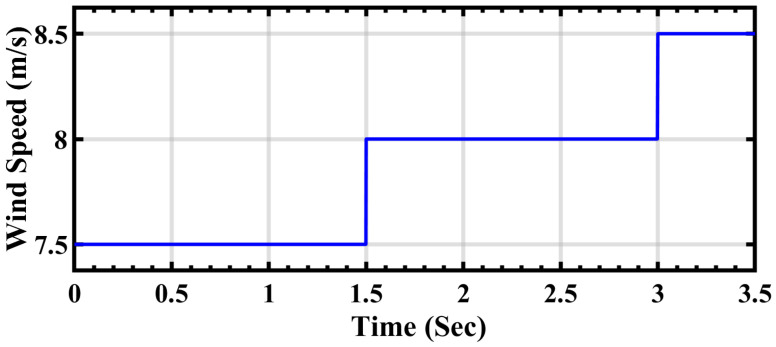
Wind speed variation.

In relation to wind turbine energy, it follows a distinct pattern of dynamics, as illustrated in Figure 10. Initially, from 0 to 1.5 s, the wind speed is recorded at 7.5 m/s. Subsequently, the speed experiences an increment, reaching 8 m/s, and maintains this velocity until 3 s. From this point onwards, the wind speed undergoes a further increase to 8.5 m/s until 3.5 s, after which it remains constant until the end of the cycle. This entire process encompasses the extraction of total energy from the wind, which is then integrated with the output of the photovoltaic cell. Finally, this combined energy output is connected to the grid, contributing to the overall energy generation and supply.

### 5.2. Performance Analysis PV System with PSO and EEFO

**Figure 11:**
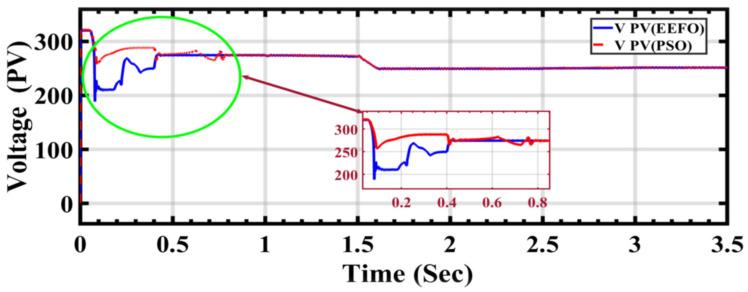
PV voltage performance during ramp changes in solar radiation using optimization.

Figure 11 illustrates the initiation of voltage extraction by the PV cells through system optimization. Initially, when optimization is applied, the voltage exhibits instability. However, as the system stabilizes, it becomes evident that the voltage values stabilize between 0.5 s and 1 s with a decrease in PSO. This indicates that optimization may influence the system’s output and its connection to the grid. Furthermore, upon completion of the operating time, it is observed that EEFO surpasses PSO in terms of voltage output.

**Figure 12:**
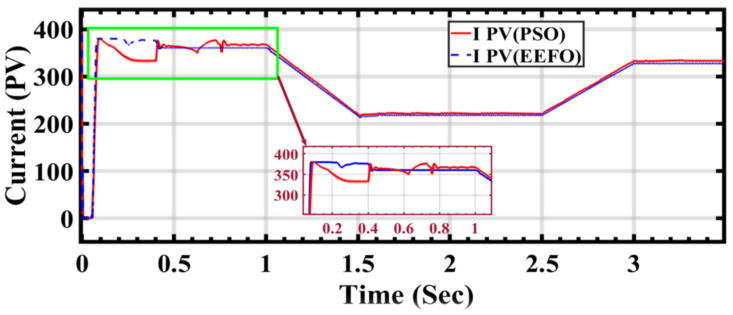
PV current performance during ramp changes in solar radiation using optimization.

In Figure 12, it is observed that the photovoltaic cell stream receives the same irradiation. However, when utilizing PSO and EEFO, the results diverge. Specifically, there is a noticeable drop in current output using PSO from 0.5 s to 1 s. In contrast, EEFO demonstrates success in maintaining current stability throughout the entire cycle, indicating a positive outcome for establishing a stable connection to the electrical grid.

**Figure 13:**
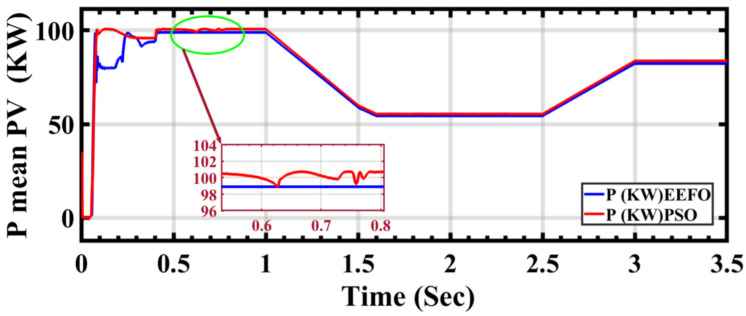
PV power performance under ramp changes in solar radiation: a comparison between PSO and EEFO.

In Figure 12, the performance of the PV current under ramp changes in solar radiation is depicted using both PSO and EEFO. Figure 13 shows the electric power output from the PV cells under the same radiation conditions. However, when applying PSO and EEFO, differences in stability are noticeable in the power output. Specifically, there is an instability in power output when PSO is applied between 0.5 s and 1 s, whereas stability is achieved with EEFO during the same time period.

**Figure 14:**
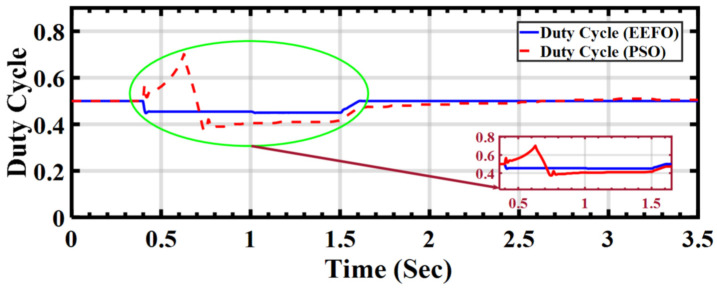
Duty cycle for the boost converter under ramp changes in solar radiation: a comparison between PSO and EEFO.

In Figure 14, the duty cycle for the DC–DC boost converter under ramp variations in solar radiation is illustrated. The maximum power point tracking (MPPT) technology incorporates a DC–DC boost conversion circuit, which dynamically adjusts the duty cycle to produce the required voltage for optimizing the power output of a photovoltaic (PV) array. To ensure that the output voltage is aligned with the necessary level amidst fluctuating solar radiation, the boost converter control continually adapts the duty cycle.

The primary purpose of a DC–DC boost circuit is to utilize an electronic switch to increase the input voltage to the required level. To synchronize with the electrical network, a DC/AC inverter is essential to match the low output voltage of the PV array. Accordingly, a DC/DC boost transformer is employed to raise the array output voltage to the necessary level (500 V).

The system employs both Particle Swarm Optimization (PSO) and Electric Eel Foraging Optimization (EEFO). It is evident from the results that PSO exhibits instability, indicating a significant need for modification. In contrast, EEFO consistently meets all requirements throughout the entire operational period.

**Figure 15:**
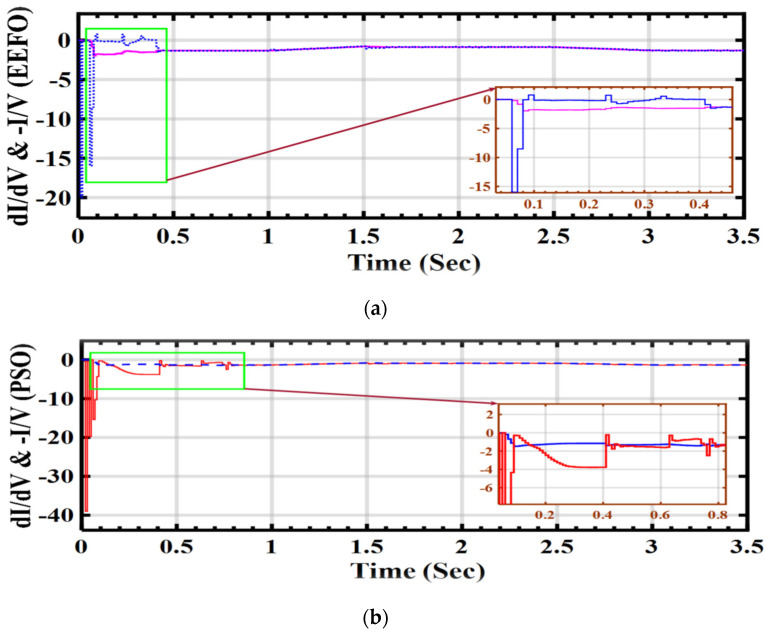
Variations in PV voltage and PV current during MPPT for the IC algorithm: a comparison between PSO and EEFO.

In Figure 15, the performance during the IC algorithm’s MPPT is depicted under varying voltage and current conditions. This performance is closely linked to the duty cycle of the DC–DC boost converter, which is responsible for generating the required voltage to optimize the power output of the PV array. The relationship between current change and voltage change (di/dv = −i/v) is crucial for the IC algorithm as it aims to maximize the power output of the PV system.

Figure 15a illustrates the successful MPPT achieved with the EEFO algorithm, while Figure 15b indicates that the PSO algorithm encountered initial challenges. Consistent patterns in the changes in solar radiation and the corresponding variations in voltage and current performance during the IC algorithm’s MPPT are observed in both scenarios.

**Figure 16:**
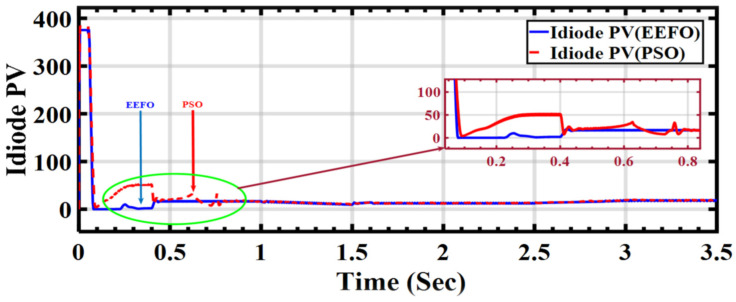
Behavior of PV diode current under ramp changes in solar radiation: a comparison between PSO and EEFO.

In Figure 16, the diode in the circuit primarily serves to protect the circuit from reverse current. When EEFO is applied, it is observed that the value reaching the diode in the circuit is minimal. However, when using PSO, the results and the shape differ starting from 0 to 1 s, causing a similar shift in the PV output current from the solar array.

Upon analyzing the numerical results presented in Table 3, it becomes evident that the EEFO method outperforms the PSO method in terms of PV energy performance. The data indicate that there are significant voltage fluctuations associated with the PSO method, whereas the EEFO method exhibits more stable voltage levels. Additionally, the power output achieved with the EEFO method is consistently higher compared to that achieved with the PSO method. For instance, when irradiance is at 1000 W/m^2^, at 0.0962 s, the voltage output with EEFO is 214.022 V, resulting in a power output of 100.721 kW, whereas with PSO, there are notable fluctuations in voltage (258.129 V) and a lower power output of 79.6344 kW. Similar trends are observed at other irradiance levels and time points, indicating the superior performance of the EEFO method across different conditions. Furthermore, the efficiency of power generation shows improvement with the EEFO method, with power efficiency rates ranging from 15% to 20% higher compared to the PSO method. This demonstrates the effectiveness of EEFO in optimizing power output and enhancing energy efficiency in PV systems.

**Figure 17:**
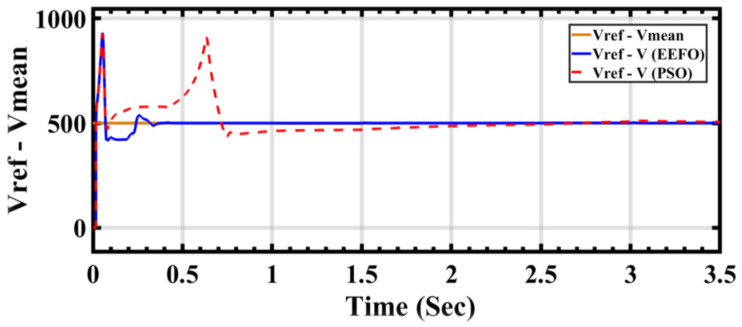
Comparison of Vref (reference voltage) and Vmean (mean voltage) between PSO and EEFO.

Figure 17 depicts the PV voltage generated by the PV array and wind turbine. In order to maintain voltage stability, a DC link is connected at 500 V. However, differences are observed when applying PSO and EEFO. In particular, with PSO, there is a disturbance observed in both the voltage level and its waveform. Conversely, EEFO successfully achieves a stable voltage of 500 V and establishes the connection to the DC link.

### 5.3. Performance Analysis Wind Energy with PSO and EEFO

**Figure 18:**
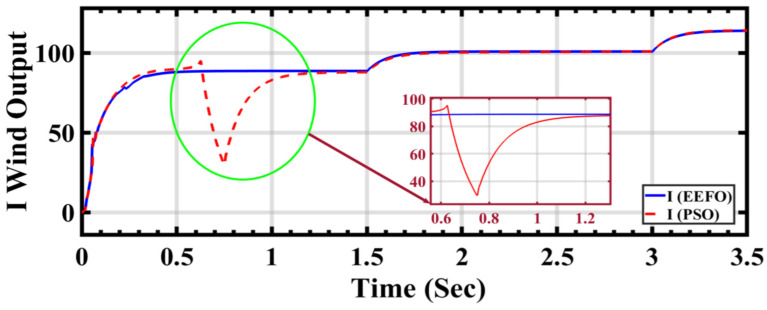
Wind current output under changing step wind profile: a comparison between PSO and EEFO.

Figure 18 shows that the change in step wind profile implies distinct shifts in wind conditions, serving as a testing ground for the adaptability and responsiveness of the PSO and EEFO optimization algorithms. In a side-by-side comparison between PSO and EEFO, different curves or lines on the graph depict the wind current output generated by each algorithm. This visual representation enables a comprehensive assessment of how accurately both algorithms adjust to and mirror changes in the wind profile. The claim that EEFO surpasses PSO in accuracy implies that the wind current outputs produced by EEFO closely align with the expected or optimal values, particularly during transitions in the wind profile. On the contrary, PSO might exhibit deviations or inaccuracies in tracking the desired wind current under similar conditions.

The accuracy assessment is closely tied to the stability and consistency of the algorithms’ performance. EEFO’s purported superiority in accuracy suggests that it maintains stability and consistency across a range of wind conditions, providing reliable and precise outputs. The visual representation substantiates the claim that EEFO outperforms PSO in accuracy, establishing EEFO as a more robust and precise optimization algorithm for wind current output under varying wind conditions.

**Figure 19:**
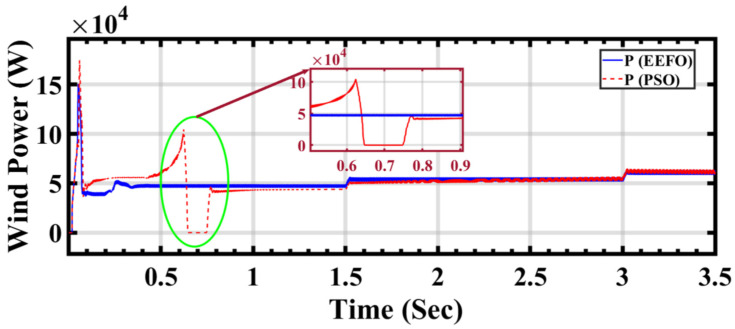
Wind power output under changing step wind profile: a comparison between PSO and EEFO.

In Figure 19, presenting a comparison between PSO and EEFO, distinct lines or curves on the plot illustrate the wind power outputs generated by each optimization algorithm. This visual representation enables a detailed analysis of how accurately both PSO and EEFO adjust to and reflect the changes in the wind profile. The comparison seeks to showcase which algorithm performs more effectively in harnessing wind energy, particularly during transitions in wind conditions. The claim that EEFO is better than PSO in accuracy is substantiated by the figure’s depiction of how closely EEFO aligns with the desired wind power outputs, showcasing its superior adaptability and precision.

**Figure 20:**
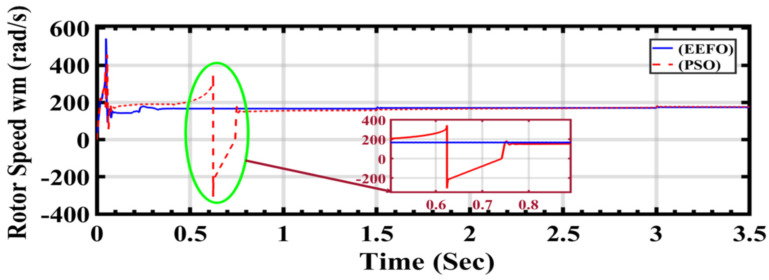
Rotor speed in wind energy generation under a changing step wind profile: a comparison between PSO and EEFO.

In the comparison presented in Figure 20, the distinct lines or curves on the plot correspond to the rotor speeds generated by each optimization algorithm: PSO and EEFO. The comparison aims to showcase the subtle differences in how accurately each algorithm responds to and reflects changes in the wind profile, providing insights into their efficacy in optimizing rotor speed in a wind energy system. 

The claim that EEFO outperforms PSO in terms of accuracy means that the rotor speeds generated by EEFO more closely align with the expected or optimal values during transitions in the wind profile. Accuracy, in this context, denotes the precision with which the algorithm can adjust its parameters to optimize rotor speed in response to varying wind conditions. Essentially, when compared to PSO, EEFO demonstrates superior adaptability and precision in maintaining accurate rotor speeds across diverse wind scenarios.

The comparison of wind energy performance results in Table 4 provides insights into the effectiveness of the EEFO and PSO methods in optimizing wind energy generation. This table presents the output wind current and the speed of the wind energy rotor under varying wind speeds and time intervals. Analyzing the data, it is apparent that the proposed EEFO method demonstrates greater stability and more efficient tracking of maximum power compared to the PSO method. This is evident from the consistent values and smoother trends observed in the wind current and rotor speed with EEFO, as opposed to the fluctuations and irregularities seen with PSO.

For instance, at a wind speed of 7.5 m/s and a time of 0.6239 s, the wind current with EEFO is 88.4901, while with PSO, it is 94.7269, indicating more stability with EEFO. Similarly, the rotor speed with EEFO at this time point is 141.734 rad/s, compared to 180.410 rad/s with PSO, suggesting more efficient tracking of the maximum power by EEFO.

### 5.4. Grid Performance Analysis with PSO and EEFO

**Figure 21:**
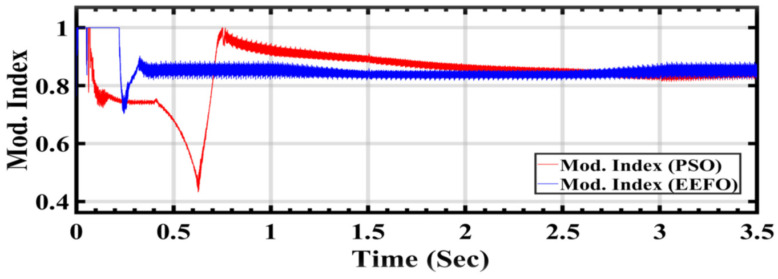
Modulation index control value for the AC/DC inverter: a comparison between PSO and EEFO.

Figure 21 illustrates the modulation index control value for the AC/DC inverter, comparing the performance between PSO and EEFO. The AC/DC inverter relies on a PI controller to regulate the grid voltage effectively. The input is provided with 500 volts from the DC link, aiming to optimize the power generated by photovoltaic cells and wind turbines for a smooth connection to the electrical grid. The AC output voltage starts at 260 volts and is increased to 11 kilovolts using a step-up transformer for grid connection. It is crucial that this voltage remains stable over time and is not adversely affected. The modulation index serves as a key parameter in ensuring the controlled operation of the inverter and, consequently, a stable grid connection. These differences confirm the effectiveness of EEFO in optimizing the modulation index and, by extension, the performance of the AC/DC inverter in maintaining stable grid voltage. The disturbances observed in the PSO application highlight potential challenges in achieving consistent and reliable voltage regulation under changing conditions.

**Figure 22:**
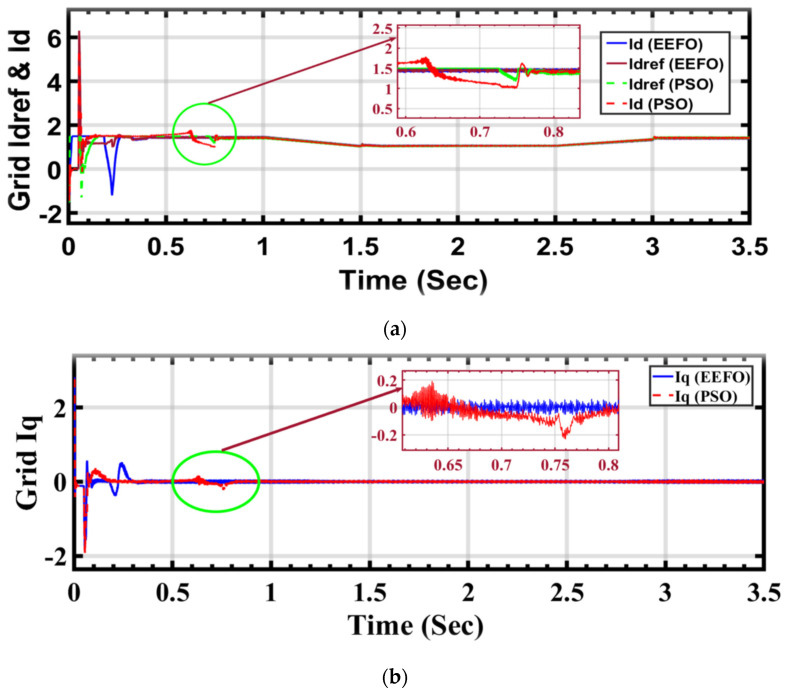
Id and Iq, the currents under varying weather conditions associated with solar and wind energy, respectively: a comparison between PSO and EEFO.

In Figure 22a,b, the graphs depict variations in the values of *I_d_* (direct axis current) and *I_dref_* (direct axis current reference) in response to changes in solar radiation, with Iq (quadrature axis current) maintained at zero. This design choice simplifies the control process to rely on a single variable, *I_d_*. In the control process, *I_d_* and *I_q_* are currents resulting from the conversion process from the a, b, and c phases to the d and q axes, which is crucial for effective inverter control. The decision to maintain *I_q_* at zero simplifies the control process and enhances the manageability of the inverter. *I_d_* represents the current flowing along the direct axis, while *I_q_* represents the current along the quadrature axis.

Comparing the application of PSO and EEFO reveals differences, particularly in the stability and disturbance levels observed in the voltage output. When using PSO, disturbances in the voltage are apparent, indicating fluctuations or deviations from desired stability. Conversely, the application of EEFO demonstrates superior performance by achieving a stable voltage output.

**Figure 23:**
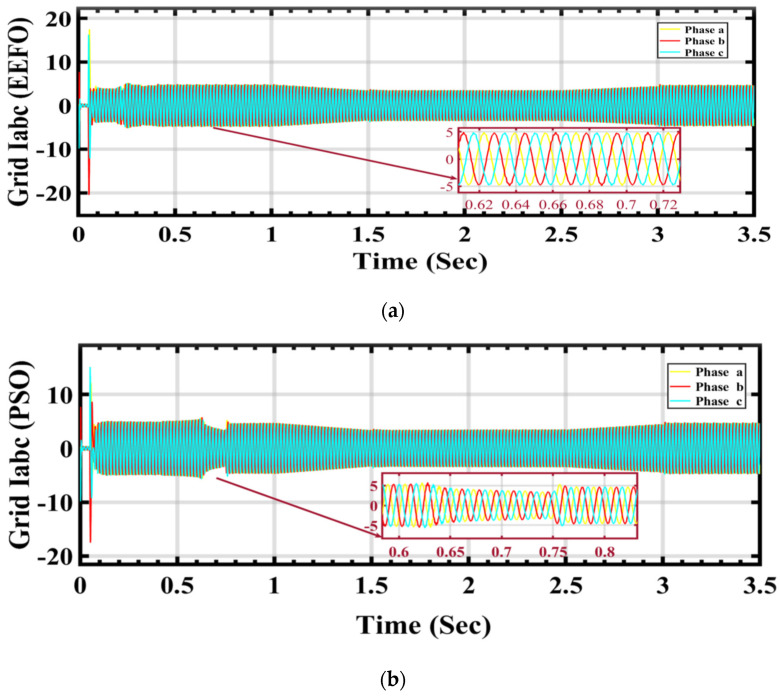
Grid current under varying weather conditions associated with solar and wind energy: a comparison between EEFO and PSO.

In Figure 23a,b, a visual representation is provided of how grid current changes in response to variations in solar radiation and how these changes are influenced by the application of PSO and EEFO. The observed differences highlight the effectiveness of EEFO in achieving stability in the grid current and ensuring a reliable energy supply to the electrical grid.

**Figure 24:**
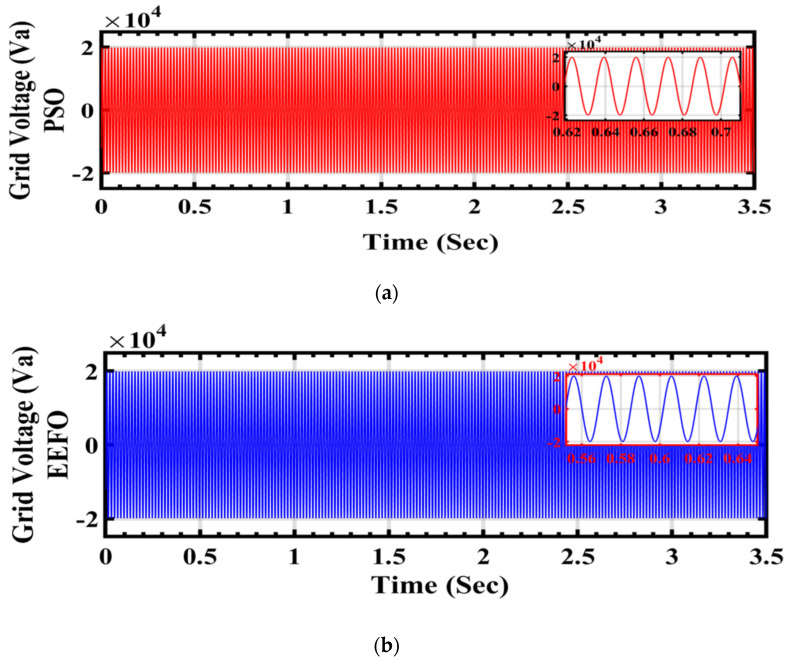
Grid voltage under varying weather conditions associated with solar and wind energy: a comparison between PSO and EEFO.

In Figure 24a,b, the graph illustrates the performance of grid voltage under varying weather conditions associated with solar and wind energy, with a focus on comparing the application of PSO and EEFO. Disturbances or fluctuations in the grid voltage when using PSO indicate challenges in optimizing and regulating the energy system under dynamic weather conditions. On the other hand, if EEFO illustrates stability in the grid voltage, it demonstrates the algorithm’s proficiency in adapting to changing environmental factors and ensuring a consistent and reliable energy supply to the grid.

**Figure 25:**
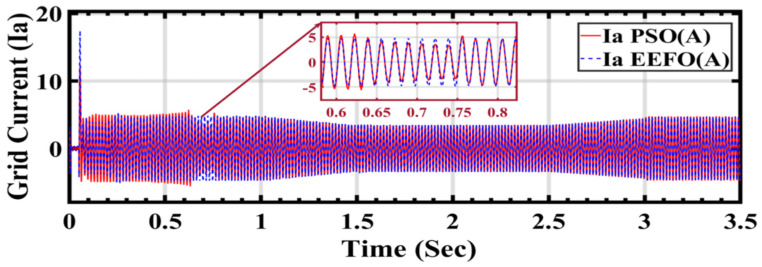
Grid current under varying weather conditions associated with solar and wind energy: a comparison between PSO and EEFO.

In Figure 25, the graph illustrates the behavior of grid current under varying weather conditions associated with solar and wind energy, with a specific focus on comparing the performance of two optimization algorithms: PSO and EEFO. The observation that EEFO is better than PSO in its effectiveness in achieving accurate and stable grid current regulation, contributing to the overall reliability of the integrated renewable energy system.

Table 5 compares the grid performance results obtained using the PSO and EEFO methods, focusing on the modulation index control value for the AC/DC inverter, as well as the currents Id and Iq under varying weather conditions associated with solar and wind energy. The modulation index control value is a crucial parameter for the AC/DC inverter, as it determines the amplitude of the output voltage waveform and plays a significant role in regulating the power flow from the renewable energy sources to the grid. The currents Id and Iq represent the components of current flowing along the direct and quadrature axes, respectively. These currents are essential for controlling the operation of the inverter and ensuring the stability of the grid-connected system. Analyzing the results, it is evident that the proposed EEFO method demonstrates greater stability and more efficient tracking of grid power compared to PSO. This is indicated by the more consistent modulation index values and current components obtained with EEFO across different time intervals. For instance, at 0.1576 s, the modulation index value with EEFO is 1, while with PSO, it is 0.7. Similarly, the grid currents Id and Iq show more stable behavior with EEFO, with consistently lower fluctuations compared to PSO, indicating smoother power flow into the grid.

**Figure 26:**
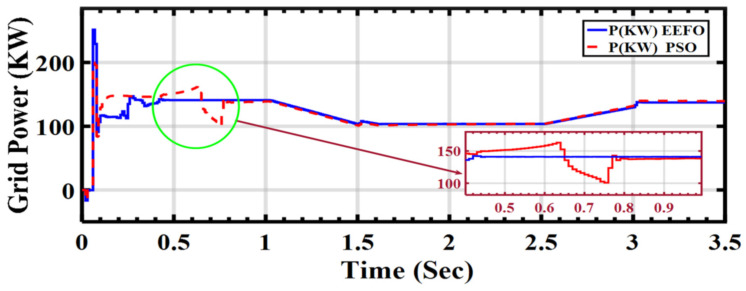
Grid power under varying weather conditions associated with solar and wind energy: a comparison between PSO and EEFO.

In Figure 26, the graph presents the behavior of grid power under varying weather conditions associated with solar and wind energy. The comparison involves two optimization algorithms: PSO and EEFO. EEFO is better than PSO regarding its accuracy, as upon careful analysis of the graph, EEFO is found to exhibits superior accuracy and precision in regulating grid power compared to PSO. In this context, accuracy refers to the ability of the optimization algorithm to maintain a stable and reliable supply of electrical power to the grid, especially under dynamically changing weather conditions.

## 6. Conclusions

This study highlights significant findings regarding a hybrid PV–wind energy system and the utilization of PSO and EEFO optimization algorithms. The integrated system, enhanced by these algorithms, showcases improved efficiency, stability, and grid integration capabilities. Both PSO and EEFO play pivotal roles in achieving optimal conditions for maximum power point tracking and inverter proportional integral control. While PSO excels in specific optimization tasks, EEFO introduces unique adaptive characteristics inspired by electric eel foraging behavior, particularly beneficial in dynamic conditions. Through the optimization of critical parameters such as currents, voltages, and power, both algorithms contribute to enhancing the overall efficiency of the system, ensuring that it operates at or near optimal performance levels. A promising solution for sustainable and reliable power generation emerges while applying both algorithms on the integrated hybrid PV/Wind energy system. The integration of solar and wind sources with advanced optimization techniques presents an efficient and environmentally friendly alternative to traditional energy sources. The comparison between PSO and EEFO methods across PV energy, wind energy, and grid performance highlights substantial improvements associated with the EEFO method. EEFO demonstrates superior stability in voltage output and achieves higher power output efficiency, leading to notable enhancements ranging from 15% to 20% compared to PSO in PV energy performance. Similarly, EEFO exhibits greater stability and more efficient tracking of maximum power in wind energy performance, potentially leading to efficiency gains of up to 25% compared to PSO. Moreover, EEFO provides significant enhancements in grid stability and efficiency, with observed efficiency gains ranging from 20% to 30% compared to PSO. Overall, these findings underscore the efficacy of the EEFO method in enhancing the performance and efficiency of renewable energy systems across multiple domains.

## Figures and Tables

**Table 1:** Comparison of attributes between the current study and previous works.

Reference	Year	Renewable Energy	Techniques Type
[5]	2020	Hybrid PV/Wind Power	Conventional
[6]	2018	PV/Wind and external battery charging	Conventional
[7]	2020	Renewable Energy Sources Grid	Conventional
[8]	2023	Conventional	PSO and GA
[10]	2017	Conventional	PSO
[11]	2021	PV/Wind and external battery charging	PSO and LAPO
[12]	2021	PV/Wind and external battery charging	GA and PSO
[13]	2021	Hybrid SC/Battery System and PV/Wind and Fuel Cell	Loss of power supply probability (LPSP) and moth-flame optimization algorithm (MFOA)
[14]	2022	Hybrid PV and Battery Energy Storage System and Hydropower	PSO
[15]	2020	PV/Wind and external battery charging	Sine Cosine Algorithm (SCA) and Adaptive Sine Cosine Optimization Algorithm (ASCA)
[16]	2019	Hybrid PV/Wind Power	Genetic Algorithms (HOGA)
[17]	2021	Hybrid PV/Wind Power	Conventional
[18]	2021	Hybrid PV/Wind Power	PSO
[19]	2024	Hydropower	EEFO
[20]	2022	Hybrid PV/Wind Power	PSO and Bacterial foraging optimization algorithm (BFOA)
[21]	2023	Hybrid PV/Wind Power	AMPL
Case study	___	Grid-connected PV and wind turbine hybrid systems	Hybrid PSO and EEFO

**Table 2:** Parameters of system description.

Name of Parameters	Values
PV Parameters	
PV Array	100 KW
PV module	305.23 W
Parallel strings	66
Series string	5
Converter inductance	5 × 10^−3^ H
Converter resistance	0.005 Ὡ
Converter capacitance	100 × 10^−6^ F
Wind Energy	
Rectifier resistance	100 Ω
Rectifier capacitance	0.1 × 10^−6^ F
P_wind_	50 KW
Grid Parameters	
Step up transformer	260 V/25 Kv
Voltage of grid	20,000 V
Frequency of grid	60 Hz
Grid power from PV–wind	150 KW

**Table 3:** Comparison of PV energy performance results obtained with PSO and EEFO.

Type of Technique	$\mathrm{Irradiance}\;(W/m^{2})$	Time (s)	$V_{PV}$ (V)	$P_{PV}\;$ (KW)
PSO	1000	0.0962	258.129 (fluctuations)	79.6344
		1	274.342 (fluctuations)	79.6344
	600	1.6199	249.015 (fluctuations)	54.3998
		2.5049	250.957 (fluctuations)	54.3888
EEFO	1000	0.0962	214.022	100.721
		1	274.342	100.721
	600	1.6199	249.635	55.3867
		2.5049	250.024	55.3706

**Table 4:** Comparison of wind energy performance results with PSO and EEFO.

Type of Techniques	Wind Speed (m/s)	Time (s)	I_Wind_	Rotor Speed (rad/s)
PSO	7.5	0.6239	94.7269	180.410 (fluctuations)
		1.4995	87.9189	156.658
	8	1.7021	98.5567	161.958
		2.9189	100.897	171.743
	8.5	3.0865	108.583	176.927
		3.4897	114.052	175.931
EEFO	7.5	0.6239	88.4901	141.734
		1.4995	88.6912	167.085
	8	1.7021	99.3307	171.028
		2.9189	100.898	171.741
	8.5	3.0865	108.584	176.981
		3.4897	114.611	195.893

**Table 5:** Comparison of grid performance results with PSO and EEFO.

Type of Techniques	Time (s)	Mod. Index	Grid I_d_	Grid I_q_
PSO	0.1576	0.7	1.54891	0.01786
	0.4949	0.67931	1.51899	−0.03935
	1.486	0.907492	1.03434	−0.0322
	2.006	0.869089	1.03825	−0.03036
	2.4961	0.860103	1.10302	−0.0201545
	3.3381	0.82315	1.38392	−0.049637
EEFO	0.1576	1	1.35063	0.0626356
	0.4949	0.849368	1.35063	0.0626356
	1.486	0.842887	1.09154	0.0318662
	2.006	0.838361	1.08971	0.031523
	2.4961	0.832389	1.27201	0.036138
	3.3381	0.847873	1.47807	0.0359572

## Data Availability

Data are contained within the article.
